# Hepatitis B Virus in Gabonese Non-Human Primate: Potential Zoonotic Circulation and Long-Term Strain Persistence

**DOI:** 10.3390/pathogens15050528

**Published:** 2026-05-14

**Authors:** Danielle S. Koumba Mavoungou, Larson Boundenga, Sonia E. Lekana-Douki, Neil M. Longo Pendy, Schedy E. Koumba Moukouama, Linda Bohou Kombila, Gabriel Falque, Joa Braïthe Mangombi, Augustin Mouinga-Ondeme, Vladimir Dedkov, Laurent Dacheux, Avelin F. Aghokeng, Nadine N’dilimabaka

**Affiliations:** 1 Unité Emergence des Maladies Virales, Centre Interdisciplinaire de Recherches Médicales de Franceville (CIRMF), Franceville BP 769, Gabon; s_lekana@yahoo.fr (S.E.L.-D.); koumbaschedy@gmail.com (S.E.K.M.); bohoukombilalinda@gmail.com (L.B.K.); gabfalque@hotmail.fr (G.F.); joa.mangombi@gmail.com (J.B.M.); 2 Ecole Doctorale Régionale d’Afrique Centrale en Infectiologie Tropicale (EDR), Franceville BP 876, Gabon; 3 Unité de Recherche en Ecologie de la Santé (URES), Centre Interdisciplinaire de Recherches Médicales de Franceville (CIRMF), Franceville BP 769, Gabon; boundenga@gmail.com (L.B.); longo2michel@gmail.com (N.M.L.P.); 4 Département D’anthropologie, Université de Durham, South Road, Durham DH1 3LE, UK; 5 Unité des Infections Rétrovirales et Pathologies Associées, Centre International de Recherche Médicales de Franceville (CIRMF), Franceville BP 769, Gabon; ondeme@yahoo.fr; 6 St. Petersburg Pasteur Institute, Federal Service for Consumer Rights Protection and Human Well-Being Surveillance, 197101 St. Petersburg, Russia; vgdedkov@yandex.ru; 7 Martsinovsky Institute of Medical Parasitology, Tropical and Vector Borne Diseases, Sechenov First Moscow State Medical University, 119991 Moscow, Russia; 8 Institut Pasteur, Université Paris Cité, Environment and Infectious Risks Unit, 75724 Cedex 15 Paris, France; laurent.dacheux@pasteur.fr; 9 Centre de Recherche sur les Maladies Infectieuses et Leurs Vecteurs (MIVEGEC), Université de Montpellier, Centre National de la Recherche Scientifique (CNRS), Institut de Recherche pour le Déveleppement (IRD), 34000 Montpellier, France; avelin.aghokeng@ird.fr; 10 Département de Biologie, Faculté des Sciences, Université des Sciences et Techniques de Masuku (USTM), Franceville BP 901, Gabon; 11 Ecole des Sciences et Médecine Vétérinaires de Masuku (ESMVM), Université des Sciences et Techniques de Masuku (USTM), Franceville BP 901, Gabon

**Keywords:** hepatitis B virus strain, non-human primates, chimpanzee, gorilla, little monkey, Gabon

## Abstract

Orthohepanaviruses are viruses that infect a number of mammals, including humans and non-human primates. However, previous studies in great apes in Gabon in 2001 found one strain of hepadnavirus (HBV ChBassi strain) displaying genetic recombination between human, gorilla and chimpanzee strains, suggesting cross-species transmissions among primates. The aim of this study was to evaluate the presence of HBV DNA in non-human primates (NHPs) and to compare it with human HBV strains in order to assess the zoonotic potential. We analyzed feces from 1891 NHPs, collected in forests in Gabon, to find human HBV-related hepadnaviruses by amplifying a portion of the S gene using hemi-nested techniques, followed by sequencing. A total of 51 samples were PCR-positive. Thirteen of the fourteen sequences obtained after sequencing were phylogenetically more closely related to chimpanzee HBV strains, while the fourteenth sequence was associated with the ChBassi HBV strain. This study shows that HBV infection is endemic in wild-born great apes in Gabon. The detection of a strain genetically close to the Bassi strain (a potential zoonotic strain) highlights the need for more in-depth studies to provide an effective response as part of the ‘One Health’ initiative.

## 1. Introduction

Hepatitis B virus (HBV), the prototype species of the *Orthohepadnavirus* genus in the *Hepadnaviridae* family, is a small, enveloped DNA virus with a partially double-stranded genome containing between 3182 and 3221 nucleotides [1]. HBV infection represents a major public health problem on a global scale. Indeed, HBV is distributed worldwide, and over a third of the world’s population carries serological markers of past or current infection [2]. According to the World Health Organization (WHO), hepatitis B caused around 1.1 million deaths in 2022, mainly from cirrhosis or hepatocellular carcinoma [3].

In highly endemic countries, the frequency of infection can be maintained by vertical transmission from mother to child or by horizontal transmission, particularly in early childhood [4]. Because of the morbidity and mortality associated with this infection, the WHO has set a target of eliminating viral hepatitis by 2030. This elimination of viral hepatitis is defined as reducing deaths by 65%, reducing new infections and mother-to-child transmission, and increasing vaccination coverage by 90% [5].

Field studies have shown that *Orthohepadnavirus* (HBV) also infects other mammalian species, including rodents [6,7,8], bats [9,10,11,12] and non-human primates (NHPs) [13,14,15]. The NHPs in which HBV has been isolated are chimpanzees and gorillas in sub-Saharan Africa [13,14], gibbons and orangutans in Southeast Asia [16,17], the woolly monkey in America [18], the capuchin monkey in Latin America [15] and macaques in Western Indian Ocean region [19], with prevalences that can reach those observed in humans. Although the viruses are phylogenetically close, infection of humans with HBV genotypes from NHPs has not yet been described. However, an NHP can carry both human HBV and HBV genotypes from other NHP species, illustrating the potential of primate HBV to cross the species barrier [20]. Indeed, studies performed in chimpanzees in Gabon over 20 years ago revealed the existence of a particular HBV strain (Ptt-chBassi) which is a result of a recombination between human, gorilla and chimpanzee strains, reinforcing proof of inter-species transmission of HBV strains between these three primates and indicating a possibility of human infection by an NHP strain. It is therefore necessary to monitor the prevalence of HBV infection in NHP populations, as well as circulating genotypes, in endemic areas.

Gabon is considered an endemic region for human HBV infection, with a hepatitis B surface antigen (HBsAg) prevalence above 7% [21]. Because of the possibility of zoonotic transmission, investigating HBV infection in NHP is critical to assessing this transmission risk. Currently, limited information is available on the prevalence of HBV infection in NHPs in Gabon. To try to fill this gap, in this study, we investigated and characterized NHP HBV strains and their burden in the country, knowing that a high prevalence among NHPs enhances the possibility of zoonotic transfer to humans.

## 2. Materials and Methods

### 2.1. Study Area and Sample Origin

Retrospective non-human primate samples collected as part of previous work at the Centre Interdisciplinaire de Recherches Médicales de Franceville (CIRMF) [22] were used in this study. These samples were collected during the dry and rainy seasons between 2009 and 2013 in 6 out of the 9 provinces of Gabon, namely Estuaire, Haut-Ogooué, Ngounié, Ogooué-Ivindo, Ogooué-Lolo and Woleu-Ntem. The GPS coordinates are shown in Table S1.

Sampling NHPs is difficult for ethical and technical reasons, so we used non-invasive samples consisting of feces of NHPs living in the Gabonese wilderness, collected as described previously [22]. All NHPs were genetically identified as previously described [23].

### 2.2. Sample Preparation and DNA Extraction

The semi-liquid fecal samples were preserved in RNAlater. Due to the limited availability of materials, fecal samples were grouped into pools containing ten monospecific species at most. DNA was extracted from 200 µL of liquid feces using the commercial QIAamp Fast DNA Stool Mini kit (Qiagen, Hilden, Germany) according to the manufacturer’s recommendations.

### 2.3. Polymerase Chain Reaction and Sequencing

DNA extracts were used for hemi-nested PCR. A portion of the S gene (376 bp) was amplified using specific primers. The primers for the first PCR (shown below from 5′ to 3′ direction) were HBV2853P TCACCATATTCTTGGGAACA and HBV409N AGATGAGGCATAGCAGCAGGATG. The primers for the hemi-nested PCR were HBV58P CCTGCTGGTGGCTCCAGTTC and HBV409N [24]. Invitrogen Platinum *Taq* DNA Polymerase (Thermo Fisher Scientific, Waltham, MA, USA) was used for the first and second rounds of PCR at volumes of 0.4 μL and 0.1 μL, respectively. The composition of the reaction mix for the first run was a follow: 12.5 μL of 2× buffer, 0.04 μg of bovine serum albumin (BSA), 0.4 μM of each primer (sense and antisense), 4 µL of molecular biology water, 0.5 µL of enzyme and 5 μL of DNA. The reaction mix for the second round consisted of 2.5 μL of 10× buffer, 0.02 μg of BSA, 0.2 mM of dNTP, 1.5 mM of MgCl_2_, 0.4 μM of each primer, 17.65 µL of molecular biology water, 0.1 µL of enzyme, and 1 μL of amplicon from the first round. The final volume for both the first and second rounds was 25 μL.

The PCR program for the first round was as follows: 5 min at 94 °C, followed by 40 cycles of 30 s at 94 °C, 1 min at 55 °C and 1 min at 72 °C, with a final elongation step at 72 °C for 2 min. For the second round, it was 5 min at 94 °C, followed by 40 cycles of 30 s at 94 °C, 30 s at 55 °C and 30 s at 72 °C, with a final elongation step at 72 °C for 2 min. During these analyses, molecular biology water was used as negative control, while the positive control was an NHP sample positive for HBV.

After PCR, 1.5% agarose gel electrophoresis was performed, and PCR products were visualized under UV light using Quantum-ST4 1100/26MX software (Vilber Lourmat, 77202 Marne La Vallée, France). Sample pools with bands of the expected size (376 bp) were considered positive. Samples from the positive pools were then tested separately. PCR-positive samples were sequenced on the ABI Prism 3500 genetic analyzer (Applied Biosystems, Thermo Fisher Scientific, Illkirch-Graffenstaden, France) using the BigDye Terminator V1.1 Cycle Sequencing Kit (Applied Biosystems, Foster City, CA, USA) as previously described [12].

### 2.4. Statistical Analysis

A LASSO (Least Absolute Shrinkage and Selection Operator) regression was performed to identify the most important predictors of HBV infection. The LASSO technique, which is a regularization method, was chosen to handle potential multicollinearity and reduce overfitting by imposing a penalty on the regression coefficients. The analysis was carried out using the “cv.glmnet” function from the glmnet package in R (version 4.4.0), which performs cross-validation to determine the optimal regularization parameter (lambda). The model was fitted using a binomial family to model the binary outcome of HBV infection (HBV = 1 vs. HBV = 0). The predictors included in the model were Years, Season, Gender, Area, and Category. The resulting coefficients were interpreted to assess the impact of each variable on the odds of HBV infection. Positive coefficients indicate an increase in the odds of infection, while negative coefficients indicate a decrease. Proportions between groups were compared using Chi-squared tests (chisq.test) when the assumptions for expected frequencies were met. In cases where expected counts were too low or the table was larger than 2 × 2, Fisher’s exact test (fisher.test) with simulated *p*-values was used to ensure validity. For comparisons involving more than two groups, post-hoc pairwise comparisons were performed using the fisher.multcomp function from the RVAideMemoire package, with Bonferroni correction applied to control for multiple testing.

### 2.5. Phylogenetic Analysis

The sequences were first assembled using ChromasPro software version 1.7.7 (Technelysium Pty, Ltd., Cheltenham, Victoria, Australia) and then aligned with other NHP and human HBV sequences (surface gene) from GenBank using MEGA 11 software. The phylogenetic tree was estimated from the alignment of 376 nucleotides (accession numbers and national origins of each strain are indicated on the tree) using MEGA 11 software. The maximum likelihood method based on the Tamura–Nei model was used to perform the phylogenetic analysis with 1000 bootstrap replicates.

## 3. Results

A total of 1891 non-human primate feces samples were analyzed. Among them, over half (1051 (55.6%)) were from chimpanzees, while the remainder were from gorillas (705 (37.3%)) and small monkeys (135 (7.1%)) (Table 1 and Table S1). The small monkey population consisted mostly of *Mandrillus sphinx* 67 (49.6%) and *Colobus satanas* 33 (24.4%). The remaining small monkey species were *Cercopithecus cephus*, *Cercopithecus nictitans*, *Cercopithecus solatus*, *Lophocebus albigena* and *Papio anubis*, with less than four individuals per species. Nearly half of the feces samples, 838 (44.3%), were collected in national parks, and the rest, 1053 (55.7%), were collected in community forests. Also, most samples were collected in the province of Ogooué-Ivindo, where the number of chimpanzee samples, 451 (48.9%), and gorilla samples, 404 (43.9%), were practically equivalent. In contrast, in the province of Haut-Ogooué, three-quarters of the samples came from chimpanzees (501 (77.3%)). Most of these samples were collected during the rainy season (1716 (90.7%)) (Table 1).

### 3.1. HBV Detection in Non-Human Primates

**Figure 1:**
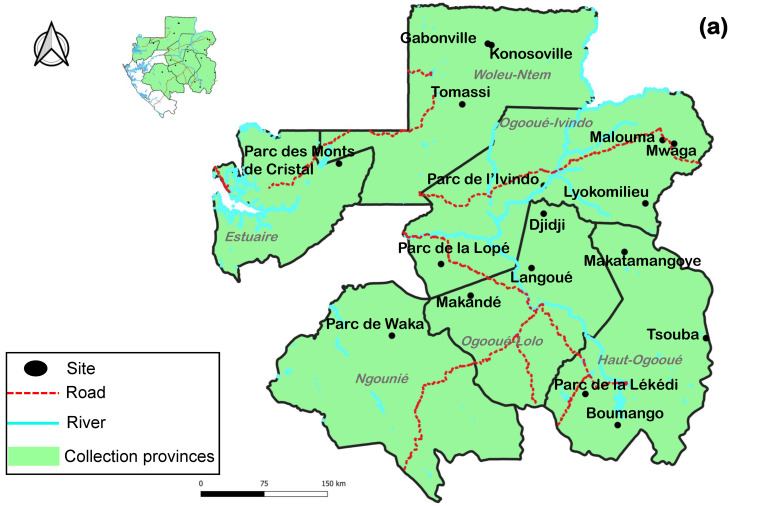

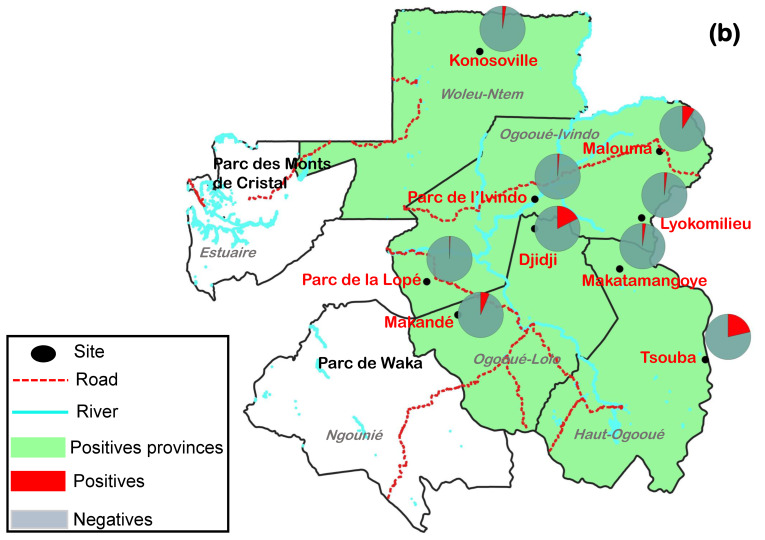
(**a**) Distribution of non-human primates in the six sampling provinces. Black dots represent collection sites. (**b**) Distribution of primate HBV PCR-positive samples by collection site. Gray pie charts represent PCR-negative samples and red pie charts represent PCR-positive samples.

Out of the 1891 feces samples, 221 pools were created and tested. Of these pools, 39 were PCR-positive, with 51 individual samples showing DNA fragments at the expected size after hemi-nested PCR and agarose gel electrophoresis, giving an overall prevalence of 2.7%. Of the positive samples, 42/1891 (2.2%) were from chimpanzees, while 9/1891 (0.5%) were from gorillas. No small apes were found positive for HBV. There were 46/1053 (4.4%) HBV-positive samples in community forests, while 5/838 (0.6%) samples in national parks were positive (Table 1). The number of HBV-positive feces samples in Lopé and Ivindo national parks was 3/838 and 2/838, respectively (Table S2). As for HBV-positive feces samples collected in community forests, 3/1053 and 13/1053 came from the Tsouba and Makatamangoye forests in Haut-Ogooué, respectively, 1/1053 and 14/1053 came from the Lyokomilieu and Malouma forests in Ogooué-Ivindo, respectively, 3/1053 and 10/1053 came from the Makandé and Djidi forests in Ogooué Lolo, respectively, and finally 2/1053 came from Konosoville in Woleu-Ntem (Table S2). The rest of the national parks and forests yielded no HBV-positive samples (Table 1 and Table S2, Figure 1b). The number of positive samples varies from year to year. The lowest prevalence was 0.4% (1/257) in 2009, while the highest was 5.9% (22/370) in 2011. No positive samples were found in 2013. The prevalence of HBV-positive samples in the dry season, 12/175 (6.9%), was higher than in the rainy season at 39/1716 (2.9%) (*p* ≤ 0.0004998) (Table 1).

### 3.2. Phylogenetic Analysis

**Figure 2:**
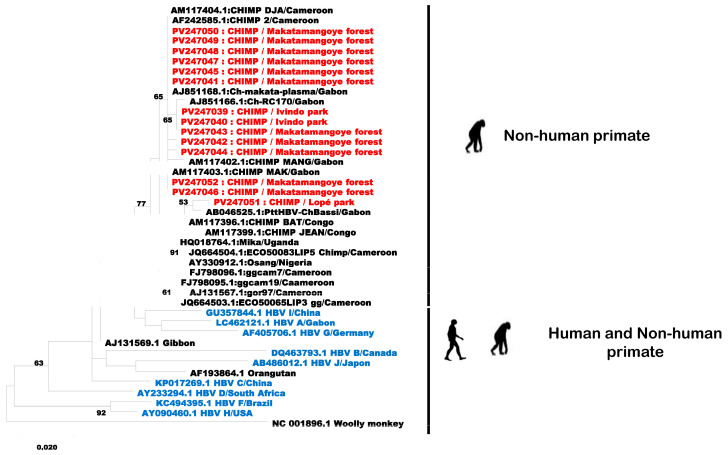
Evolutionary analysis by Maximum Likelihood method. The evolutionary history was inferred by using the Maximum Likelihood method and Tamura–Nei model. The tree with the highest log likelihood (−1419.38) is shown. The percentage of trees on which the associated taxa clustered together is shown next to the branches. Initial tree(s) for the heuristic search were obtained automatically by applying Neighbor-Join and BioNJ algorithms to a matrix of pairwise distances estimated using the Tamura–Nei model and then selecting the topology with superior log likelihood value. The tree is drawn to scale, with branch lengths measured in the number of substitutions per site. This analysis involved 42 nucleotide sequences. Codon positions included were 1st + 2nd + 3rd + Noncoding. There was a total of 316 positions in the final dataset. Evolutionary analyses were conducted in MEGA X. Sequences from this study are shown in red, while sequences from previous studies uploaded to GenBank are shown in black for non-human primates and in blue for humans.

Of the 51 PCR-positive samples, 16 were sequenced and 14 sequences were obtained. Alignment was performed with a portion of the S gene (376 bp) from these 14 sequences and others available on GenBank, using the NCBI BLAST “BLASTn” algorithm. According to phylogenetic analysis based on a portion of the Orthohepadnavirus S gene, the NPH HBVs clustered into four monophyletic clades. The first clade (six sequences) included HBV sequences from chimpanzees isolated in Cameroon and Gabon, while the second and the third (seven sequences) included HBV sequences from chimpanzees isolated only in Gabon. The last clade was grouped with a sequence previously isolated in Gabon (Figure 2). Of the 14 sequences, 3 were isolated from the feces of chimpanzees living in Ivindo (n = 2) (accession number PV247039 and PV247040) and Lopé (n = 1) national parks (accession number PV247051), while the remaining 11 were isolated from chimpanzee feces collected in Makatamangoye forest (PV247041 to PV247050, PV247052). All the sequences related to our samples were isolated from chimpanzees born in the wild but living in captivity. The sequences obtained in our study had a nucleotide identity between 99% and 100% with those present in the literature. The non-human primate sequences found in this study were related only to other non-human primate sequences available in GenBank.

### 3.3. Factors Favoring HBV Positivity in Non-Human Primates

We compared the proportions of HBV between the different primate species, with the variables of area, year and season of collection, to determine the parameters influencing the occurrence of infection. First, we found that chimpanzees (*p*-value = 8.239 × 10^−6^) and gorillas (*p*-value = 3.383 × 10^−2^) living in community forests were more infected than gorillas living in national parks (Table S3). Second, the stool samples of chimpanzees collected in 2012 were significantly more infected with respect to gorilla samples from 2009 and 2010 (*p*-value < 0.001) (Table S4). Third, chimpanzee samples collected during both the dry and rainy seasons were more infected than gorilla samples collected during the rainy season (Table S5).

The LASSO results show that the variables community forest (coefficient = 2.34) and chimpanzee species are the most important predictors of HBV occurrence in NHP. In fact, the analysis reveals that in the community forests, HBV infection is more frequent, whereas in national parks, infection is less frequent. In addition, the coefficient of −0.97 means that chimpanzees are more affected by HBV infection than gorillas. The coefficient value of −1.1 for the rainy season shows that the dry season could have an impact on the increase in the prevalence of infection. Only the variables community forest and chimpanzee species significantly increase the probability of HBV infection (Table 2).

## 4. Discussion

In this study, a large panel of samples (1891) of non-human primate feces collected in six (Estuaire, Haut-Ogooué, Ngounié, Ogooué-Ivindo, Ogooué-Lolo and Woleu-Ntem) of the nine provinces of Gabon were analyzed to detect hepatitis B virus (HBV). HBV was detected in 51 NHP fecal samples, of which 42 (4%) were from chimpanzees and 9 (1.3%) were from gorillas, giving an overall prevalence of 2.7% (51/1891). The prevalence of HBV DNA found in our study is higher than that obtained by Makuwa and colleagues, who did not detect HBV DNA in feces [25]. Another study by Makuwa and colleagues found a similar prevalence (2.2%) of HBV DNA in Gabonese wild chimpanzees [24]. Although we obtained a prevalence of less than 3%, numerous studies have described HBV prevalences in NHPs like those recorded in human populations in areas of high endemicity. Indeed, another study by Makuwa and colleagues conducted in great apes from Gabon and Congo described seroprevalences of infection as high as 32%, with DNA detected in over 4% of NHPs [25]. Other studies carried out on great apes in Cameroon have obtained HBV DNA detection rates ranging from 6% to 18% in plasma [13,26]. The difference in prevalence between these studies can be explained by the sample size, which is much larger in our study than in previous ones. However, the nature of the samples can also explain these differences. Indeed, viral DNA contained in feces is sometimes subject to degradation, with low viral titers compared to blood and liver samples.

In our study, the number of infection-positive chimpanzee feces samples (42/1051) is higher than that of gorillas (9/705) (*p*-value = 0.0015). The same observations have been made in studies carried out in great apes from Gabon, Cameroon and Congo [13,25,26,27].

This result could be explained by the fact that chimpanzees may be more susceptible to HBV infection. Indeed, numerous studies have used chimpanzees as animal models to investigate the pathogenesis of HBV-induced diseases and to test new antiviral therapies [28]. However, this disparity in prevalence could also be attributed to notable differences in social behaviors specific to each species. Chimpanzees are territorial and will not hesitate to physically confront their neighbors to defend their territory [29]. In addition, chimpanzees live in dense social communities characterized by a multi-male–multi-female structure, promoting frequent and complex interactions between individuals. Gorillas, on the other hand, adopt a more restricted social organization, typically single-male–multi-female, with lower group density and more limited inter-individual contact [30]. The dense social structure of chimpanzees is conducive to increased intra-group aggression and close physical contact, thereby facilitating the transmission of pathogens that are transmitted through contact with bodily fluids, such as HBV. Furthermore, their polygynous–polyandrous mating system (simultaneous polyandry and polygyny) increases the likelihood of sexual transmission of the virus compared to gorillas, whose reproductive behavior is more monopolistic, with a single dominant male having access to the females in the group [31].

We also looked for the presence of molecular markers in small monkeys of the family *Cercopithecidae*. No traces of HBV DNA were detected. The latest studies looking for HBV in this family of NHPs by Makuwa and colleagues obtained similar results [25,27]. Another study carried out in Cameroon on *Cercopithecidae* monkeys showed HBsAg seroprevalence of around 3%. However, given the low optical densities obtained in the ELISA tests, these results could be due to a non-specific reactivity; the authors refrain from making any definitive conclusions about the occurrence of HBV infection in small monkeys [32]. Studies revealing HBV DNA in *Cercopithecidae* are rare. Dupinay et al. and Lu et al. detected HBV DNA in *Macaca fascicularis* and *Rhinopithecus roxellana* [19,33]. The difference observed among these studies and ours can be explained, on one hand, by the nature of the samples. Indeed, Dupinay and Lu used serum and liver samples, which would have higher viral loads. On the other hand, it can be explained by the fact that the animals tested in Dupinay and Lu’s studies were captive animals, unlike ours, which were wildlife. In addition, at the moment, only small monkeys from Asia, America and the Indian Ocean region have been found positive for HBV DNA, suggesting that some monkey species may not be susceptible to HBV infection [15,19,33]. However, the small number of African monkey samples used in all the studies can also be a limiting factor.

The prevalence of HBV in great apes appears to have increased over the years from 0.4% in 2009 to 1.8% in 2010 and 5.9% in 2011, although it fell in 2012 (3.2%), and no positive samples were recorded in 2013. The overall analysis shows a significant difference in infection depending on the year. However, when the parameters were analyzed two by two, this difference only concerns chimpanzees from 2012 and gorillas from 2009 and 2010, suggesting that the infection is stable and endemic over time. Nevertheless, the negative figures for 2013 can be explained by the small size of the sample. In addition, the prevalence of infection in the dry season was higher than in the rainy season. The difference in the prevalence of HBV infection observed between seasons could be explained by NHPs’ diet during different seasons. It has been shown that during the dry season, great apes tend to share the same food niche, increasing the frequency of contact between them [34], which leads to the increase in the frequency of pathogen’s transmission. There are no studies in the literature on the prevalence of HBV infection in non-human primates according to season that would enable comparisons with the present data. However, a previous study carried out on bats also provided evidence that HBV infection was significantly more prevalent in the dry season than in the rainy season [12].

Also, we noticed that the prevalence of HBV infection in great apes living in community areas (4.4%) is higher than that observed in national parks (0.6%). Unfortunately, once again, there is no data in the literature to enable comparisons of HBV infection in chimpanzees living in national parks and those living in community forests. Nevertheless, human activities such as hunting in community forests cause permanent stress in great apes, leading to an increase in the production of cortisol, which results in immune deficiency, making them susceptible to infection [35,36]. This could explain why more chimpanzees from community forests are infected than those from national parks.

The study of the predictors of HBV infection in great apes in Gabon shows that an increased risk of HBV infection in apes is found in chimpanzees living in community forests. This result is not surprising, as most chimpanzee feces were collected in community forests (766/1053, compared to 285/838 in parks). However, other factors like social behaviors specific to each species and the immune response deficiency due to permanent stress, as mentioned above, may also explain this susceptibility of chimpanzees from community forests.

Phylogenetic analyses based on a portion of the S gene revealed that the 14 HBV sequences isolated from chimpanzee feces are closely related to the chimpanzee (*Pan troglodytes*) HBV strains previously isolated in Gabon, Cameroon and Congo, with nucleotide identity percentages close to 100% no matter the season or year of sample collection. These results could suggest that a single strain of ChHBV circulates predominantly in the chimpanzee population in Gabon. However, the size of the analyzed fragment does not allow us to draw any conclusions. Indeed, HBV is classified into genotypes and subgenotypes on the basis of a nucleotide divergence of 8% and 4%, respectively, depending on whether we are dealing with a complete genome or surface gene analysis only [37]. Thus, we can conclude that no new HBV strains were identified in this study.

However, of the 14 sequences, 1 was closely related to the Bassi strain (accession number AB046525.1), an HBV strain isolated from a chimpanzee of the *Pan troglodytes troglodytes* subspecies with a percentage identity of 99.19%. The complete genome of the Bassi strain showed a nucleotide difference of between 9% and 26% compared with those reported to date in primates. In addition, it was shown that during routine serological tests, the Bassi strain was positive for hepatitis B virus surface antigen (HBsAg). HBsAg positivity could be attributed to the similarity of its amino acid sequence with all circulating HBV genotypes, highlighting a similarity with human strains. In view of these observations, the authors suggested that this was a new strain detected in central Africa [38]. It was first detected in 2001 and has not since been isolated from other primates in Gabon or neighboring countries, probably due to the paucity of studies. Our results, however, suggest that this strain is probably still circulating in great apes in Gabon.

The particularity of the Bassi strain is that it is the product of recombination of three strains: human HBV strains of genotypes G and E, a gorilla strain (GoHBV, accession number AJ131567) and a chimpanzee strain (ChHBV, accession number AF222322), in the pre-S1 domain [38]. The HBV Pre-S1 domain has been shown to play a key role in HBV infectivity. It is responsible for the binding of the virus to the host cell (hepatocyte) [39]. Modifications in the sequence of the Pre-S1 domain could, therefore, modulate the infectivity of the virus, as has been previously demonstrated [40]. Further, zoonotic transmission from a non-human primate to a human could be possible. However, due to the size of our sequence, we were unable to identify any recombination event supporting this idea. Indeed, the recombination recorded in the different strains of HBV was highlighted by phylogenetic analysis of the complete genome sequences of human and non-human HBV [41,42]. Numerous studies have demonstrated genetic recombination between (i) human HBV strains of different genotypes (genotypes A and D, A and C, B and C or between D and C [41]), (ii) chimpanzee and human HBV strains [42], and (iii) chimpanzee and gorilla strains [26]. These data reinforce the hypothesis of a zoonotic transmission of HBV strains between NHPs and humans.

The Gabonese strain related to the Bassi strain was isolated from the feces of a chimpanzee living in the Lopé National Park. This result underlines the potential risk posed by these primates to human populations that can be in contact with them. Indeed, depending on the availability of food, animals can move between national parks and community forests. In addition, many tourists visit this national park every year, and villagers living around the park frequent it, enhancing both the possibility of contact with NHPs carrying the Bassi strain and the possibility of zoonotic transmission of this strain to humans. Thus, there is a need for more in-depth studies on the circulation of this strain in and around Lopé National Park to provide an effective response as part of the ‘One Health’ initiative to contribute to the elimination of HBV by 2030.

## 5. Conclusions

In conclusion, the results of this study complete those of a previous study, research conducted by Makuwa and collaborators on great apes, and show both active circulation of chHBV in NHPs in Gabon and the endemic character of this infection. Although HBV strains from HNPs have never been found in humans, this could change. Indeed, the detection of the Bassi HBV strain, a recombination of human, gorilla and chimpanzee strains that is able to infect humans, highlights a potential risk for human infection and zoonotic transfer of HBV from chimpanzees to humans. One of the limitations identified in this study was that we were unable to sequence all PCR-positive samples due to a lack of funding and make the complete genomes of the strains identified. This enabled a deep comparative analysis with sequences from the literature to see if there is any HBV genetic diversity in the great apes of Gabon and to further explore in the potential zoonotic transmission of the similar Bassi strain to humans. It would also have been interesting to compare the genetic sequences of HBV strains detected in human populations with those of NHPs living in the same regions. This would have enabled a better assessment of the potential zoonotic risk. Another limitation of this study was the use of feces to test for HBV DNA. It would also have been interesting to look for serological markers of HBV infection to determine the real burden of this infection in NHPs in Gabon. In addition, we were unable to determine the exact prevalence of the Bassi strain in our study population because we did not sequence all the samples due to limited reagent resources.

## Figures and Tables

**Table 1:** Characteristics of the study population and PCR positives.

Variables		Nb. Positive PCR/Total nb.				Fisher Test/Chi Test (*p*-Value)
		Chimpanzee	Gorilla	Little Monkey	Total	
Areas						
	National park	4/285	1/440	0/113	838	
	Community forests	38/766	8/265	0/22	1053	<0.0001
Years						
	2009	0/9	1/204	0/44	257	
	2010	7/171	1/182	0/84	437	
	2011	15/297	7/71	0/2	370	<0.0001
	2012	20/397	0/226	0/5	628	
	2013	0/177	0/22	0	199	
Seasons						
	Dry	5/71	7/80	0/24	175	0.0017
	Rainy	37/980	2/625	0/111	1716	
Total		42/1051	9/705	0/135	51/1891	0.0015

**Table 2:** Selected variables and their coefficients by LASSO in the HBV infection prediction model.

Variable	Coefficient	Interpretation
Intercept	1015.25	Base log-odds
Years	−0.56	Reduction in odds per year
Season (Rainy)	−1.13	Reduced odds during the rainy season
Gorilla Gender	−0.97	Reduced odds in the presence of gorilla
Community forest	2.34	Increased odds in community forests

## Data Availability

The original data presented in the study are openly available in [Zenodo] at https://zenodo.org/uploads/19451345 (accessed on 7 March 2026).

## Supplementary Materials

The following supporting information can be downloaded at https://www.mdpi.com/article/10.3390/pathogens15050528/s1.

## References

[1] Bowyer S.M., Sim J.G.M. (2000). Relationships within and between genotypes of hepatitis B virus at points across the genome: Footprints of recombination in certain isolates. J. Gen. Virol..

[2] Stanaway J.D., Flaxman A.D., Naghavi M., Fitzmaurice C., Vos T., Abubakar I., Abu-Raddad L.J., Assadi R., Bhala N., Cowie B. (2016). The global burden of viral hepatitis from 1990 to 2013: Findings from the Global Burden of Disease Study 2013. Lancet.

[3] World Health Organization (2024). Hepatitis B. https://www.who.int/news-room/fact-sheets/detail/hepatitis-b.

[4] Dumpis U., Holmes E.C., Mendy M., Hill A., Thursz M., Hall A., Whittle H., Karayiannis P. (2001). Transmission of hepatitis B virus infection in Gambian families revealed by phylogenetic analysis. J. Hepatol..

[5] World Health Organization (2021). Interim Guidance for Country Validation of Viral Hepatitis Elimination.

[6] Tyler G.V., Summers W., Snyder R.L. (1981). Woodchuck hepatitis virus in natural woodchuckpopulations. J. Wildl. Dis..

[7] Summers J., Smolec J.M., Snyder R. (1978). A virus similar to human hepatitis B virus associated with hepatitis and hepatoma in woodchucks. Proc. Natl. Acad. Sci. USA.

[8] Marion P.L., van Davelaar M.J., Knight S.S., Salazar F.H., Garcia G., Popper H., Robinson W.S. (1986). Hepatocellular carcinoma in ground squirrels persistently infected with ground squirrel hepatitis virus. Proc. Natl. Acad. Sci. USA.

[9] Drexler J.F., Geipel A., König A., Corman V.M., Van Riel D., Leijten L.M., Bremer C.M., Rasche A., Cottontail V.M., Maganga G.D. (2013). Bats carry pathogenic hepadnaviruses antigenically related to hepatitis B virus and capable of infecting human hepatocytes. Proc. Natl. Acad. Sci. USA.

[10] Wang B., Yang X.-L., Li W., Zhu Y., Ge X.-Y., Zhang L.-B., Zhang Y.-Z., Bock C.-T., Shi Z.-L. (2017). Detection and genome characterization of four novel bat hepadnaviruses and a hepevirus in China. Virol. J..

[11] Yang L., Wu J., Hu T., Qin S., Deng B., Liu J., Zhang F., He B., Tu C. (2018). Genetic diversity of bat orthohepadnaviruses in China and a proposed new nomenclature. Infect. Genet. Evol..

[12] Koumba Mavoungou D.S., Bohou Kombila L., Longo Pendy N.M., Koumba Moukouama S.E., Lekana-Douki S.E., Maganga G.D., Leroy E.M., Aghokeng A.F., N’dilimabaka N. (2024). Prevalence and Genetic Diversity of Bat Hepatitis B Viruses in Bat Species Living in Gabon. Viruses.

[13] Njouom R., Mba S.A.S., Nerrienet E., Foupouapouognigni Y., Rousset D. (2010). Detection and characterization of hepatitis B virus strains from wild-caught gorillas and chimpanzees in Cameroon, Central Africa. Infect. Genet. Evol..

[14] Makuwa M., Souquière S., Bourry O., Rouquet P., Telfer P., Mauclère P., Kazanji M., Roques P., Simon F. (2007). Complete-genome analysis of hepatitis B virus from wild-born chimpanzees in central Africa demonstrates a strain-specific geographical cluster. J. Gen. Virol..

[15] de Carvalho Dominguez Souza B.F., König A., Rasche A., de Oliveira Carneiro I., Stephan N., Corman V.M., Roppert P.L., Goldmann N., Kepper R., Müller S.F. (2018). A novel hepatitis B virus species discovered in capuchin monkeys sheds new light on the evolution of primate hepadnaviruses. J. Hepatol..

[16] Warren K.S., Heeney J.L., A Swan R., Verschoor E.J. (1999). A New Group of Hepadnaviruses Naturally Infecting Orangutans (*Pongo pygmaeus*). J. Virol..

[17] Norder H., Ebert J.W., Fields H.A., Mushahwar I.K., Magnius L.O. (1996). Complete sequencing of a gibbon hepatitis B virus genome reveals a unique genotype distantly related to the chimpanzee hepatitis B virus. Virology.

[18] Lanford R.E., Chavez D., Brasky K.M., Burns R.B., Rico-Hesse R. (1998). Isolation of a hepadnavirus from the woolly monkey, a new World primate. Proc. Natl. Acad. Sci. USA.

[19] Dupinay T., Gheit T., Roques P., Cova L., Chevallier-Queyron P., Tasahsu S.-I., Le Grand R., Simon F., Cordier G., Wakrim L. (2013). Discovery of naturally occurring transmissible chronic hepatitis B virus infection among macaca fascicularis from mauritius island. Hepatology.

[20] Rasche A., Souza B.F.D.C.D., Drexler J.F. (2016). Bat hepadnaviruses and the origins of primate hepatitis B viruses. Curr. Opin. Virol..

[21] Groc S., Abbate J.L., Le Gal F., Gerber A., Tuaillon E., Albert J.L., Nkoghé D., Leroy E.M., Roche B., Becquart P. (2019). High prevalence and diversity of hepatitis B and hepatitis delta virus in Gabon. J. Viral Hepat..

[22] Boundenga L., Ollomo B., Rougeron V., Mouele L.Y., Mve-Ondo B., Delicat-Loembet L.M., Moukodoum N.D., Okouga A.P., Arnathau C., Elguero E. (2015). Diversity of malaria parasites in great apes in Gabon. Malar. J..

[23] Santiago M.L., Lukasik M., Kamenya S., Li Y., Bibollet-Ruche F., Bailes E., Muller M.N., Emery M., Goldenberg D.A., Lwanga J.S. (2003). Foci of Endemic Simian Immunodeficiency Virus Infection in Wild-Living Eastern Chimpanzees (*Pan troglodytes schweinfurthii*). J. Virol..

[24] Makuwa M., Souquière S., Clifford S.L., Mouinga-Ondeme A., Bawe-Johnson M., Wickings E.J., Latour S., Simon F., Roques P. (2005). Identification of hepatitis B virus genome in faecal sample from wild living chimpanzee (*Pan troglodytes troglodytes*) in Gabon. J. Clin. Virol..

[25] Makuwa M., Souquière S., Telfer P., Leroy E., Bourry O., Rouquet P., Clifford S., Wickings E.J., Roques P., Simon F. (2003). Occurence of hepatitis viruses in wild-born non-human primates: A 3 year (1998–2001) epidemiological survey in Gabon. J. Med. Primatol..

[26] Lyons S., Sharp C., LeBreton M., Djoko C.F., Kiyang J.A., Lankester F., Bibila T.G., Tamoufé U., Fair J., Wolfe N.D. (2012). Species association of hepatitis b virus (HBV) in non-human apes; evidence for recombination between gorilla and chimpanzee variants. PLoS ONE.

[27] Makuwa M., Souquière S., Telfer P., Bourry O., Rouquet P., Kazanji M., Roques P., Simon F. (2006). Hepatitis viruses in non-human primates. J. Med. Primatol..

[28] Wieland S.F. (2015). The Chimpanzee Model for Hepatitis B Virus Infection. Cold Spring Harb. Perspect. Med..

[29] Morrison R.E., Dunn J.C., Illera G., Walsh P.D., Bermejo M. (2020). Western gorilla space use suggests territoriality. Sci. Rep..

[30] Grueter C.C., Chapais B., Zinner D. (2012). Evolution of Multilevel Social Systems in Nonhuman Primates and Humans. Int. J. Primatol..

[31] Walsh P.D., Breuer T., Sanz C., Morgan D., Doran D., Doran-Sheehy D. (2007). Potential for Ebola Transmission between Gorilla and Chimpanzee Social Groups. Am. Nat..

[32] Foupouapouognigni Y., Mba S.A.S., Njouom R. (2011). Prevalence of hepatitis B virus infection among Cercopithecidae monkeys in Cameroon. J. Med. Primatol..

[33] Lu G., Pan J., Zhang Y., Sun X., Ou J., Ji J., Yin X., Li S. (2021). Hepatitis B virus detected in a golden monkey fatal case, China. Infect. Genet. Evol..

[34] Roscelin L., Tédonzong D., Willie J., Tagg N., Tchamba M.N., Evaristus T., Myriane A., Keuko P., Petre C.A. (2019). The distribution of plant consumption traits across habitat types and the patterns of fruit availability suggest a mechanism of coexistence of two sympatric frugivorous mammals. Ecol. Evol..

[35] Carlitz E.H.D., Miller R., Kirschbaum C., Gao W., Hänni D.C., Van Schaik C.P. (2016). Measuring hair cortisol concentrations to assess the effect of anthropogenic impacts on wild chimpanzees (*Pan troglodytes*). PLoS ONE.

[36] Garber P.A., McKenney A., Bartling-John E., Bicca-Marques J.C., de la Fuente M.F., Abreu F., Schiel N., Souto A., Phillips K.A. (2020). Life in a harsh environment: The effects of age, sex, reproductive condition, and season on hair cortisol concentration in a wild non-human primate. PeerJ.

[37] Kurbanov F., Tanaka Y., Mizokami M. (2010). Geographical and genetic diversity of the human hepatitis B virus. Hepatol. Res..

[38] Takahashi K., Mishiro S., Prince A.M. (2001). Novel hepatitis B virus strain from a chimpanzee of Central Africa (*Pan troglodytes troglodytes*) with an unusual antigenicity of the core protein. Intervirology.

[39] Herrscher C., Roingeard P., Blanchard E. (2020). Hepatitis B Virus Entry into Cells. Cells.

[40] Iwamoto M., Tsukuda S., Watashi K., Matsuda M. (2022). N-terminal PreS1 Sequence Regulates Efficient Infection of Cell Culture-generated Hepatitis B Virus. Hepatology.

[41] Yang J., Xi Q., Deng R., Wang J., Hou J., Wang X. (2007). Identification of Interspecies Recombination Among Hepadnaviruses Infecting Cross-Species Hosts. J. Med. Primatol..

[42] Magiorkinis E.N., Magiorkinis G.N., Paraskevis D.N., Hatzakis A.E. (2005). Re-analysis of a human hepatitis B virus (HBV) isolate from an East African wild born Pan troglodytes schweinfurthii: Evidence for interspecies recombination between HBV infecting chimpanzee and human. Gene.

