# Supplementary material for: Hepatitis B Virus in Gabonese Non-Human Primate: Potential Zoonotic Circulation and Long-Term Strain Persistence
Source: Pathogens. 2026 May 14;15(5):528. doi: 10.3390/pathogens15050528 (PMC13209310; doi:10.3390/pathogens15050528)
Supplement: Supplementary file 1 [file pathogens-15-00528-s001.zip › Table S1.pdf]

Table S1. List of collection sites and geographical coordinates in Gabon

Variables		Collection sites	Coordinates	
Provinces				
	Estuaire	Parc national des Monts de cristal	0.65416N	10.61277E
	Haut-Ogooué	Boumango	-2.06643N	13.51483E
		Makatamangoye	-0.26527N	13.58583E
		Parc national de la Lékédi	-1.74361N	13.17861E
		Tsouba	-1.162367N	14.43398
	Ngounié	Parc national de Waka	-1.13638N	11.16388E
	Ogooué-Ivindo	Malouma	0.89916N	13.98194E
		Mwaga	0.86166N	14.10222E
		Lyokomilieu	0.24N	13.8033E
		Parc national d'Ivindo	0.424867N	12.74598E
		Parc national de la Lopé	-0.39138N	11.67555E
	Ogooué-Lolo	Djidji	0.13295N	12.74418E
		Langoué	-0.43333N	12.61805E
		Makandé	-0.71944N	11.98388E
	Woleu-Ntem	Gabonville	1.9022N	12.16222E
		Konosoville	1.8875N	12.2005E
		Tomassi	1.2702778N	11.89666E
