# Supplementary material for: Hepatitis B Virus in Gabonese Non-Human Primate: Potential Zoonotic Circulation and Long-Term Strain Persistence
Source: Pathogens. 2026 May 14;15(5):528. doi: 10.3390/pathogens15050528 (PMC13209310; doi:10.3390/pathogens15050528)
Supplement: Supplementary file 1 [file pathogens-15-00528-s001.zip › Table S2.pdf]

**Table S2.** Characteristics of the study population according to collection site and PCR positives

| Variables | | Collection sites | Chimpanzee | PCR positives Gorilla | Little monkey | Total |
|---|---|---|---|---|---|---|
| **Provinces** | | | | | | |
| | Estuaire | Parc national des Monts de cristal | 0 | 0/2 | 0/5 | 7 |
| | Haut-Ogooué | Boumango | 0 | 0/5 | 0 | 5 |
| | | Makatamangoye | 13/487 | 0/60 | 0/2 | 549 |
| | | Parc national de la Lékédi | 0 | 0/37 | 0/43 | 80 |
| | | Tsouba | 3/14 | 0 | 0 | 14 |
| | Ngounié | Parc national de Waka | 0/12 | 0/33 | 0 | 45 |
| | Ogooué-Ivindo | Malouma | 14/149 | 10 | 0 | 159 |
| | | Mwaga | 0/4 | 0 | 0 | 4 |
| | | Lyokomilieu | 1/25 | 0/26 | 0/1 | 52 |
| | | Parc national d'Ivindo | 2/73 | 0/43 | 0/41 | 157 |
| | | Parc national de la Lopé | 2/200 | 1/325 | 0/24 | 549 |
| | Ogooué-Lolo | Djidji | 3/21 | 7/34 | 0/2 | 57 |
| | | Langoué | 0/1 | 0/35 | 0/2 | 38 |
| | | Makandé | 2/5 | 1/27 | 15 | 47 |
| | Woleu-Ntem | Gabonville | 0/9 | 0/33 | 0 | 42 |
| | | Konosoville | 2/49 | 0/30 | 0 | 79 |
| | | Tomassi | 0/2 | 0/5 | 0 | 7 |
| **Total** | | | 42/1051 | 9/705 | 0/135 | 51/1891 |
