# Supplementary material for: Hepatitis B Virus in Gabonese Non-Human Primate: Potential Zoonotic Circulation and Long-Term Strain Persistence
Source: Pathogens. 2026 May 14;15(5):528. doi: 10.3390/pathogens15050528 (PMC13209310; doi:10.3390/pathogens15050528)
Supplement: Supplementary file 1 [file pathogens-15-00528-s001.zip › Table S3.pdf]

Table S3. Comparison of HBV occurrence between different area

	A	B	C	D	E
B	1.0000	-	-	-	-
C	1.0000	1.0000	-	-	-
D	0.1088	**8.239e-06*** **	0.1559	-	-
E	1.0000	**3.383e-02** **	1.0000	1	-
F	1.0000	1.0000	1.0000	1	1

A :chimp_national park, B :Gorilla_national park, C :LM_national_park

D :Chimp_community forests, E : Gorilla_community forests, F : LM_community forests

(*) : $p<0.05$; (**) : $p<0.01$; (***) : $p<0.001$
