# Supplementary material for: Hepatitis B Virus in Gabonese Non-Human Primate: Potential Zoonotic Circulation and Long-Term Strain Persistence
Source: Pathogens. 2026 May 14;15(5):528. doi: 10.3390/pathogens15050528 (PMC13209310; doi:10.3390/pathogens15050528)
Supplement: Supplementary file 1 [file pathogens-15-00528-s001.zip › Table S4.pdf]

Table S4. Comparison of HBV occurrence between different years

	A'	B'	C'	D'	E'	F'	G'	H'	I'	J'	K'	L'	M'	N'
**B'**	1	-	-	-	-	-	-	-	-	-	-	-	-	-
**C'**	1	1	-	-	-	-	-	-	-	-	-	-	-	-
**D'**	1	1	1	-	-	-	-	-	-	-	-	-	-	-
**E'**	1	1	1	1	-	-	-	-	-	-	-	-	-	-
**F'**	1	1	1	1	1	-	-	-	-	-	-	-	-	-
**G'**	1	1	1	1	1	1	-	-	-	-	-	-	-	-
**H'**	1	1	1	1	1	1	1	-	-	-	-	-	-	-
**I'**	1	1	1	1	1	1	1	1	-	-	-	-	-	-
**J'**	1	**0.0005342*** **	1	1	**0.00179** **	0.07867	0.5019	0.2717	1	-	-	-	-	-
**K'**	1	1	1	1	1	1	1	1	1	1	-	-	-	-
**L'**	1	1	1	1	1	1	1	1	1	1	1	-	-	-
**M'**	1	1	1	1	1	1	1	1	1	1	1	1	-	-
**N'**	1	1	1	1	1	1	1	1	1	1	1	1	1	-
**O'**	1	1	1	1	1	1	1	1	1	1	1	1	1	1

A' :Chimp_2009, B' :Gorilla_2009, C' : LM_2009, D' : Chimp_2010, E' : Gorilla_2010, F' : LM_2020, G' : Chimp_2011, H' : Gorilla_2011, I' : LM_2011, J' : Chimp_2012, K' : Gorilla_2012, L' : LM_2012, M' : Chimp_2013, N' : Gorilla_2013, O' : LM_2013

(*) : p<0.05; (**) : p<0.01; (***) : p<0.001
