# Supplementary material for: Hepatitis B Virus in Gabonese Non-Human Primate: Potential Zoonotic Circulation and Long-Term Strain Persistence
Source: Pathogens. 2026 May 14;15(5):528. doi: 10.3390/pathogens15050528 (PMC13209310; doi:10.3390/pathogens15050528)
Supplement: Supplementary file 1 [file pathogens-15-00528-s001.zip › Table S5.pdf]

Table S5. Comparison of HBV occurrence between different season

	**A”**	**B”**	**C”**	**D”**	**E”**
**B”**	1.00000	-	-	-	-
**C”**	1.00000	1.00000	-	-	-
**D”**	1.00000	6.267e-01	1	-	-
**E”**	**0.00259****	**8.494e-05*****	1	**3.492e-05*****	-
**F”**	0.12405	**2.894e-02***	1	3.999e-01	1

A”: Chimp_DRY, B”: Gorilla_Dry, C”: LM_Dry, D”: Chimp_Rainy, E”: Gorilla_Rainy, F”: LM_Rainy

(*) : p<0.05; (**) : p<0.01; (***) : p<0.001
